# Dr. Charles E. Isaacs

**Published:** 1860-09

**Authors:** 


### NECROLOGICAL NOTICES.

DR. CHARLES E. ISAACS.—It is with feelings of the deepest sorrow that we are called upon to record the decease of Dr. Isaacs, whose loss will be greatly felt by the profession of this country. As a man of science, he ranked deservedly high; he was a thorough anatomist, an original and zealous investigator in physiology and pathology, and contributed largely to our medical literature. He was best known abroad and at home by his monograph on the structure and functions of the kidney, and from his researches on the pleura.

Dr. Isaacs was born in Westchester County, New York, in 1811; and at the age of twenty-one graduated in medicine at the University of Maryland. Early in life he evinced much interest in the study of the natural sciences, and cultivated, especially, botany and geology. He was appointed surgeon, during the administration of President Jackson, to accompany the Cherokees in their removal beyond the Mississippi; and in 1841 he entered the army, ranking first in his examination. He resigned his commission five years later, and, after moving about for several years, finally settled in New York, where he was soon appointed Demonstrator of Anatomy in the College of Physicians and Surgeons. He retained this post for several years, but resigned it to become Demonstrator and Adjunct Professor of Anatomy in the University Medical College. About three years ago, Dr. Isaacs moved to Brooklyn, where he died of pleuro-pneumonia, associated with Bright's disease of the kidneys, on the sixteenth of June, in the forty-ninth year of his age.

No man in the ranks of our profession was more universally beloved. In fact, it can truly be said that he left not an enemy behind him. Dr. Isaacs died too young. He saw before him an open road to eminence and fame, and with his industry, knowledge, and fixedness of purpose, had he lived, we doubt not that he would have proved to be a lasting ornament to the American medical profession.

